# Blood Investigations in Flying Men

**Published:** 1917-07-07

**Authors:** 


### BLOOD INVESTIGATIONS IN FLYING MEN.

THE effect on the human frame of mechanical advance has always been a subject of interest. Especially has this been so under two conditions—namely, when increased speed of locomotion came into question, and when the effect anticipated was injurious. At the time steam railways were introduced there were not wanting people to maintain that moving at forty miles an hour would prevent respiration. And lest we should put this down to mere ignorance and prejudice, we may recall the fact that the first motor cyclists were darkly spoken of as being likely to shake themselves into a diabetes. It is true that, now flying has come to pass, no subtle deleterious effect upon the infinitely adaptable human frame has been imputed to aviation, but this is probably simply because pioneer aviation was seen to be such a terribly dangerous thing in itself; to have such great overt risks that covert dangers were not thought of. In all probability there are no covert dangers, any more than there were in the railway compartment or in the auto-cycle. Nevertheless, it is interesting to have the results of some physiological investigations of aviators. Meyer and Seyderhelm have published in the *Deutsche Medizinische Wochenschrift* an account of blood analyses carried out on twenty-eight flying men who had been at the work for more than a year, but who had not been flying for some days at any rate before examination. Collectively the cases showed a noticeable increase of hæmoglobin and of red cells, of which all except four cases had over five million per cubic millimetre. Three cases showed nucleated red cells. The concentration of serum albumin varied within normal limits, the higher values not coinciding with high values for hæmoglobin and erythrocytes, so that these increases could not be explained as the consequences of inspissation. Another argument against such a conclusion was the general absence of leucocytosis. Lymphocytes were increased, however. The increase in hæmoglobin and in red cells was present even in aviators complaining of nervous strain; also it did not show itself in the face, peripheral vaso-constriction making some so pale as to look anæmic. The physiological changes mentioned are, of course, common in those living at high altitudes, and the authors regard them as an increased formation of new blood. But as to whether this phenomenon is caused by rarefaction of air no opinion is expressed.

